# iCanCope With Pain: Cultural Adaptation and Usability Testing of a Self-Management App for Adolescents With Persistent Pain in Norway

**DOI:** 10.2196/12940

**Published:** 2019-06-03

**Authors:** Erik Grasaas, Liv Fegran, Sølvi Helseth, Jennifer Stinson, Santiago Martinez, Chitra Lalloo, Kristin Haraldstad

**Affiliations:** 1 Department of Health and Nursing Science Faculty of Health and Sport Sciences University of Agder Kristiansand Norway; 2 Department of Nursing and Health Promotion Faculty of Health Sciences Oslo Metropolitan University Oslo Norway; 3 Child Health Evaluative Sciences The Hospital for Sick Children Toronto, ON Canada; 4 Lawrence S Bloomberg Faculty of Nursing University of Toronto Toronto, ON Canada; 5 Institute for Health Policy, Management, and Evaluation University of Toronto Toronto, ON Canada

**Keywords:** health, self-management, adolescent, chronic pain, translating, mobile app

## Abstract

**Background:**

Persistent or chronic pain is a common health problem among adolescents. Thus, it is important that they receive evidence-based strategies for symptom management. *iCanCope with Pain* is a mobile phone app designed to help adolescents cope with chronic pain. The app comprises 5 evidence- and theory-based features: (I) symptom trackers for pain, sleep, mood, physical function, and energy; (II) goal setting to improve pain and function; (III) a coping toolbox of pain self-management strategies; (IV) social support; and (V) age-appropriate pain education. The *iCanCope with Pain* app is based on theory, identified health care needs, and current best practices for pain self-management.

**Objective:**

The objectives of this study were to describe the translation and cultural adaptation of the app into the Norwegian context and evaluate the app’s usability using a phased approach.

**Methods:**

Phase 1 included translation and cultural adaptation of the app into the Norwegian context. This process used an expert panel of researchers and target group representatives who were responsible for the linguistic quality assurance and assessment. In phases 2 and 3 the app’s usability was tested. For phase 2, the assessments of usability and user experiences included observation, the think aloud method, audiovisual recordings, questionnaires, and individual interviews in a laboratory setting. For phase 3, the assessment of usability and user experience over a 2-week home-based test included questionnaires and individual end-user interviews. Overall, app usability was determined based on ease of use, efficiency, and user satisfaction. Qualitative data were analyzed using deductive content analysis. Descriptive statistics were calculated for quantitative data.

**Results:**

End users did not report any misunderstandings or discrepancies with the words or phrasing of the translated and culturally adapted app. Participants in both the laboratory- and home-based usability tests found the app self-explanatory and reported that all 5 of its features were easy to use. All tasks were completed within the allocated time frame (ie, efficiency), with few errors. Overall System Usability Scale scores were high, with average scores of 82 and 89 out of 100 from laboratory- and field-based tests, respectively. Participants liked the idea of a social support function (feature IV), although qualitative and internet server data revealed that this feature was rarely used.

**Conclusions:**

This study described the cultural and linguistic adaptation and usability testing of the Norwegian version of the *iCanCope with Pain* app. High user satisfaction, ease of use, efficiency, and only minor errors cumulatively indicated that no changes to the app were needed, with the exception of facilitating user interaction within the social support feature. The app will be used in an upcoming randomized controlled trial with a larger sample.

## Introduction

### Background

The prevalence of persistent or chronic pain in nonclinical adolescent populations is increasing and has become recognized as a growing health problem [1-3]. Chronic pain is commonly defined as pain lasting more than 3 months [4]. Previous studies have revealed high prevalence rates (approximately 15% to 35% [5-7]) of chronic pain among adolescents, which increases with age and can negatively impact all aspects of their lives. The consequences include reduced health-related quality of life and physical activity and higher risk for psychosocial problems such as stress, anxiety, and depression [8-11]. Thus, interventions focused on coping and symptom management strategies are needed to prevent adolescents’ pain conditions from continuing into their young adulthoods [12,13].

An increasing number of self-management interventions have been developed and are associated with reduced chronic pain among both children and adolescents [14]. Self-management interventions often comprise behavioral therapies and types of cognitive behavioral therapy (CBT), which may include coping skills training, imagery techniques, biofeedback, relaxation, and other symptom management strategies [15]. CBT is effective among chronic pain patients and is thus the preferred intervention for adolescents with different health disorders [16,17]. In their systematic review of the literature in this area, Fisher et al showed that self-management interventions are accessible through computer-based programs or mobile phone apps, and that such interventions may reduce chronic pain intensity among children and adolescents [15].

Adolescents are comfortable using computerized technologies and have reported that internet-delivered self-management interventions are their preferred methods for gaining information about chronic pain and pain coping skills [18,19]. However, many of the available Web-based interventions and apps have not undergone scientific evaluation. For instance, Lalloo et al [20] found a total of 279 apps that focused on pain self-management; only 8% of these had included health care professionals during their development and only 1 had undergone scientific evaluation. Thus, it is important to emphasize that adolescents should receive evidence-based content, including strategies to manage chronic pain conditions, from apps.

### The iCanCope With Pain App

The *iCanCope with Pain* app is an evidence- and theory-based pain self-management app [21] that was developed by Dr Stinson and Lalloo, in collaboration with the Centre for Global eHealth Innovation at University Health Network in Toronto, Canada. The app’s content was developed by an interdisciplinary team of pediatric chronic pain experts and is based on empirically identified health care needs and current best practices for pain self-management [21]. The app is currently part of an ongoing randomized controlled trial and is thus not publicly available.

### Theoretical Framework

**Figure 1:**
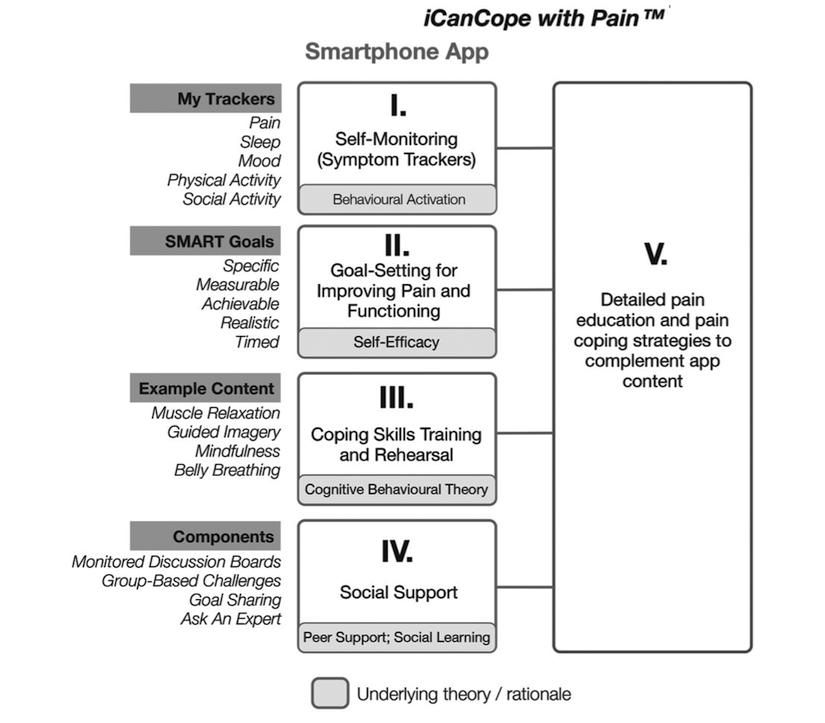
Conceptual framework showing the theories underlying the Norwegian iCanCope with Pain app for adolescents with persistent pain. Published as the original Canadian illustration. Source: Stinson et al. Used with permission by the original publishers [21].

The *iCanCope with Pain* app comprises 5 evidence- and theory-based features: (I) symptom trackers for pain, sleep, mood, physical function, and social function; (II) goal setting to improve pain and function; (III) a coping toolbox of pain self-management strategies; (IV) social support; and (V) age-appropriate pain education. Features I to IV were based on psychological theories and psychotherapies; component V is a pain education library (Figure 1).

Component I is based on behavioral activation therapy, which was originally developed to treat mood disorders and is efficacious for reinforcing engagement with, and motivation for, meaningful activity [22,23]. Allowing adolescents to track and self-monitor their daily symptoms in real time helps them to better recognize their pain patterns and set goals to improve their symptoms. It may also help them identify and be aware of their pain triggers; by tracking symptoms over time, adolescents can also monitor fluctuations in their pain [21,24]. My trackers are integrated as a daily check-in functionality in the app, wherein the adolescents can rate their level of pain intensity, pain interference, mood, physical activity, sleep quality, and energy.

Component II is based on social cognitive theory, originally called social learning theory developed by Albert Bandura, which has influenced our understanding of human behavior [25]. The theory suggests that adolescents’ performance or behavior is influenced by their beliefs (cognition) and support by their peers, parents, and teachers. Bandura argues that self-efficacy is the most suitable approach to affecting cognition [26]. Self-efficacy refers to “how well one can execute courses of action required to deal with prospective situations” [27]. Thus, component II was designed to enhance self-efficacy and thereby improve pain and functioning [19]. The development of the app’s goals feature was consistent with the SMART framework—specific, measurable, achievable, realistic, and timed [28,29]. Thorough formulation and evaluation of a goal is necessary for success; thus, this method provides a useful standardized tool for users to write and express their own goals in the app.

Component III is based on CBT, with a focus on the interrelations among thoughts, feelings, and behaviors [30]. Consistent with this, adolescents can focus on developing personal coping strategies to solve current problems and change unhelpful cognitive patterns (eg, thoughts, beliefs, and attitudes), behaviors, and emotion regulation [30]. Thus, the aim of component III is personalized self-management instruction in terms of coping skills training and rehearsal, to promote positive changes in mood, behavior, and ultimately pain itself [21]. This component provides several coping strategies to manage pain, including muscle relaxation, guided imagery, mindfulness, and abdominal breathing. In other words, the CBT component of the app aims to provide pain management strategies that help adolescents during everyday life, despite their pain [31].

Component IV, social support, includes both quantitative (eg, number of friends) and subjective (eg, network appraisal) dimensions [32], both of which affect mental health, physical health, and mortality risk, and thus influence health throughout the lifespan [33]. Social support theory and peer support are strongly related to self-efficacy (component II) and healthy activities promotion [34]. Although numerous self-efficacy promotion methods exist, Ashford et al’s review [35] showed that vicarious experience (ie, social modeling) and feedback from peers (ie, peer support) are most effective. In the social support feature in the app, the adolescents receive *questions of the day* in monitored discussion boards. Finally, component V is a pain education library, which is integrated together with the coping skills training (component III) in the app.

The primary objectives of this paper are to describe the translation and cultural adaptation of the app into the Norwegian context and to evaluate its usability using a phased approach. The phased approach assessed the translated and culturally adapted app’s usability and users’ experiences with its ease of use, efficiency, satisfaction, and sociability. An additional objective was to identify the users’ needs and technical issues, to refine the app for use in a planned prospective randomized controlled trial with a larger adolescent sample.

## Methods

### Design

**Figure 2:**
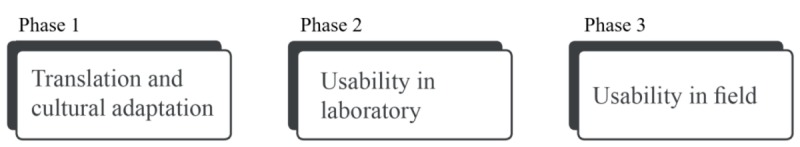
Norwegian iCanCope with Pain app translation and usability testing.

During phase 1, the *iCanCope with Pain* app was translated and culturally adapted into the Norwegian language and cultural context. This required a multistep approach, including input by an interdisciplinary group to ensure thorough translation and adaptation. During phases 2 and 3, the app’s usability was evaluated. Phase 2 was conducted in a laboratory setting and phase 3 in participants’ homes during a 2-week period. Figure 2 illustrates the overall protocol.

### Participants

Participants were recruited from a high school in Southern Norway. During phase 1, 2 representatives from the target group (both aged 17 years) participated to ensure that the app translation and cultural adaptation were appropriate for their age group. During phase 2, 6 adolescents (aged 17 to 18 years) were recruited for a laboratory-based usability test. During phase 3, 5 adolescents (aged 16 to 18 years) were recruited for a 2-week home-based test to evaluate user experiences with the app over time and to identify additional user needs. Both usability tests were gender-balanced and included users of both Android and iOS operating systems to best represent the target group for an upcoming clinical trial. The inclusion and exclusion criteria for the phase 3 end-user group were also consistent with those planned for the upcoming clinical trial. We included 16- to 19-year-old adolescents with persistent pain (weekly pain lasting 3 or more months based on subjective reporting) who were able to read and understand Norwegian and owned a mobile phone. Adolescents with cognitive disability or diseases were excluded because of their inability to correctly understand the *iCanCope with Pain* app, goal setting, or library readings. Adolescents with painful health conditions from a pathological or medical origin (eg, hematology/oncology patients) were excluded as the program was not specifically designed for these patient groups.

### Phase 1: Translation and Cultural Adaptation

A 2-stage approach was used for language and cultural adaptation of the original Canadian *iCanCope with Pain* app [21] to the Norwegian context, based on the principles of good practice for translation and cultural adaptation explained by Wild et al [36]. The first stage addressed the age-appropriate pain education library and the second stage addressed the software interface text of all features.

#### Pain Education Library

**Figure 3:**
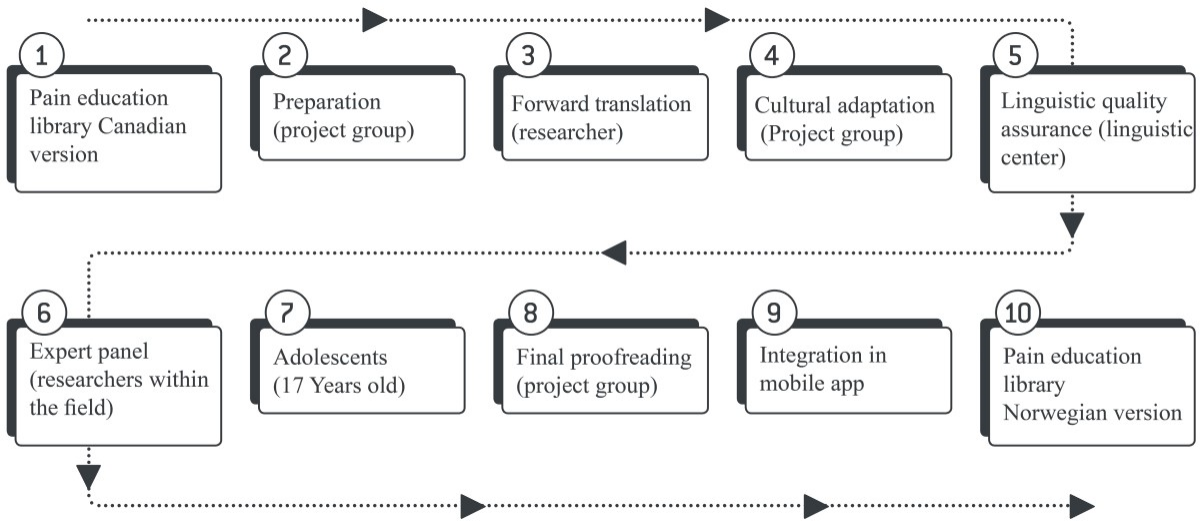
The 10 steps of translation and cultural adaptation of the iCanCope with Pain app’s pain education library.

The first stage was a 10-step process to ensure quality translation and adaptation of the age-appropriate pain education library to a Norwegian context, as illustrated in Figure 3. The first steps (1 to 4) were conducted by the project group and first author; these steps comprised preparation and forward-translation to Norwegian, followed by cultural adaptation, in which typical Canadian names, sports, and sayings were replaced with Norwegian versions (eg, dragon boat racing is not well-known in Norway). Quality assurance (step 5) was carried out by native Norwegian and English speakers at the linguistic service center at the University of Agder (UiA). In this step, the original Canadian English version was compared with the translated Norwegian version to assess linguistic equivalency and correct spelling. In addition, an expert panel of researchers within the field of pain ensured (step 6) that the 2 versions were conceptually equivalent. Furthermore, 2 adolescents assessed the pain education library (step 7) to ensure that its content was clear and easy to understand by their age group. A final proofreading (step 8) was conducted before formatting (step 9) each article in the pain education library as HTML to be added (step 10) to the Norwegian *iCanCope with Pain* app.

#### Software Interface Text

**Figure 4:**
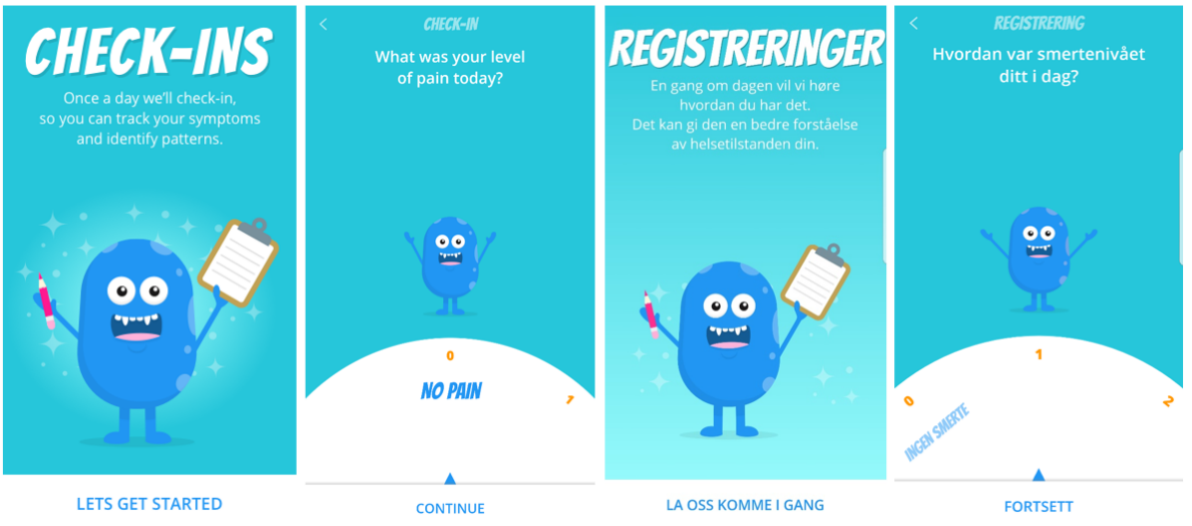
Screenshots of the Norwegian and Canadian user interface versions of the iCanCope with Pain app. Published with permission from the Centre for Global eHealth Innovation (Canada).

The second stage also followed the principles set forth by Wild et al to ensure credibility and understanding [36] and included another 10 steps: (step 1) preparation; (step 2) forward translation; (step 3) reconciliation; (step 4) back translation; (step 5) back translation review; (step 6) harmonization; (step 7) cognitive debriefing; (step 8) review of cognitive debriefing results and finalization; (step 9) proofreading; and (step 10) final report. The software interface text was prepared and translated into Norwegian by the authors (steps 1 and 2, respectively), then merged into a common version (step 3), and translated and validated back into English by personnel at the linguistic service center (steps 4 and 5, respectively). A comparison of multiple language versions was not possible as the *iCanCope with Pain* app was only available in the Canadian English language of the original version (step 6). A cognitive debriefing was conducted with the end users after the usability field test (phase 3) to check its understandability and cultural relevance (step 7). Review of the cognitive debriefing, proofreading, and final report were assessed by the project group (steps 8, 9, and 10, respectively). The Norwegian software interface text was then integrated into the *iCanCope with* *Pain* app by the Centre for Global eHealth Innovation (Canada), with adjustments to user interface size and layout to accommodate different word lengths for various screen sizes and forms. See Figure 4 for example comparisons of the Norwegian and Canadian software interfaces.

### Phase 2: Usability Test in Laboratory Setting

Before the laboratory usability test, 2 pretests were used to assess the protocol, logistics, and technology, and to determine the amount of time the tests would take, the number of participants needed, the number of tasks, and the level of app complexity. The 10 resulting predefined tasks had a stipulated time frame of approximately 1 min per task.

The task tests were carried out at the UiA laboratory facilities over 2 days with 6 adolescent participants. The laboratory facilities house control and test rooms are separated with a 1-way mirror (facility details have been previously reported by Gerdes et al) [37]. Each participant participated individually and spent approximately 60 min on research team–administered tests. Each test was conducted according to the pretested protocol, in order of: (1) 10 predefined tasks; (2) System Usability Scale (SUS) questionnaire [38]; and (3) interview. We have followed the definition by the International Organization for Standardization (ISO) by evaluating the usability in terms of the ease of use (effectiveness), efficiency, and satisfaction. The official ISO 9241-11 definition of usability is: “the extent to which a product can be used by specified users to achieve specified goals with effectiveness, efficiency, and satisfaction in a specified context of use” [39].

#### Ten Predefined Tasks

Each participant completed 10 predefined tasks corresponding to the 5 app components (Figure 1): (1) conduct a daily check-in; (2) create a goal; (3) coping skill training; (4) change goal; (5) library search; (6) create a post in the social support group; (7) complete a goal; (8) change user profile; (9) change pain area and symptoms; and (10) view history of daily check-in. The tasks were presented to each participant on a sheet of paper. Participants could ask for help at any time, in which case help was interpreted as a moderator intervention, tabulated, and annotated. Participants also performed the think aloud (TA) method [40] while solving tasks. In the TA, participants verbalize what they are thinking as they perform a task. This method is frequently used to gain insight into users’ thoughts during a usability test [40]. Observations and audio and visual recordings were collected using a set of cameras and microphones that recorded the user interface, running commentary, and physical interactions with the app. A minimum of 2 researchers were present during each test. The ease of use and technical errors were evaluated based on the number of completed tasks and total errors. A completed task was defined as a task successfully achieved by the participant [41]. An error was defined as an incorrect selection, gesture, or landing on a screen triggered by a participant. The app efficiency was evaluated based on the time needed to achieve the tasks, expressed as the mean task completion time [41].

#### System Usability Scale Questionnaire

**Figure 5:**
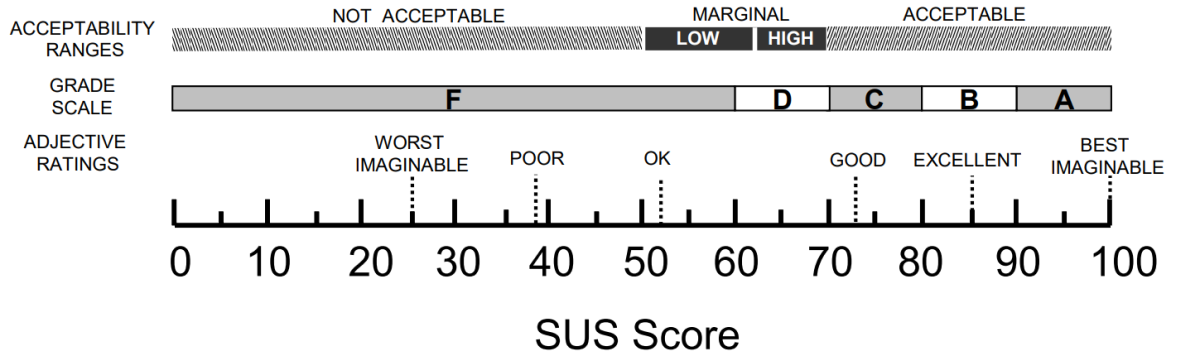
Adjective ratings, acceptability ranges, and school grading scales, in relation to the average System Usability Scale (SUS) score. Source: Bangor et al. Used with permission by the original publishers [42].

The SUS questionnaire was used to evaluate user satisfaction and comprised 10 open-ended polarity-balanced questions with a 5-point Likert scale for responses. The average scores were categorized based on the adjective ratings [42], as shown in Figure 5.

#### Interview

Finally, individual posttest semistructured interviews were conducted to assess user experiences with the app. The interview guide included 14 open-ended questions based on the 5 app components (Figure 1) and 3 additional categories for potential improvements, usage considerations, and coping. These predefined categories were considered ideal for ensuring a systematic assessment of the app and thus created a basis for the structured categorization matrix.

### Phase 3: Field Usability Test

A total of 5 adolescents with persistent pain tested the Norwegian *iCanCope with Pain* app continuously over a period of 2 weeks to assess user experience over time and to identify any need for further assistance while using the app. The participants answered an electronic survey that was equivalent to the baseline questionnaires (which will also be included in the upcoming clinical trial) to ensure that they fulfilled the inclusion criteria (eg, the Lübeck Pain questionnaire [7] for assuring the presence of pain and pain experience for 3 or more months). A detailed description of each questionnaire is available at ClinicalTrials.gov using ID NCT03551977. Each participant received an email with their username and password and an accompanying brief written introduction to the app’s features. Participants were also given a researcher’s phone number and email address, in case they needed technical assistance at any time during their 2-week participation. Participants were asked to download the app from the App Store or Google Play for their iOS- or Android-based mobile phone, respectively, after which they were to start the app and log in. User experience was assessed at the end of the 2 weeks using the SUS questionnaire and individual semistructured interviews.

### Data Analysis

The data collected (eg, internet server data, observation, audiovisual recordings, and interviews) corresponded to the 5 app components. Quantitative laboratory usability test (eg, task completion, time, errors) measures were evaluated based on users’ interactions with the app and to assess the app’s ease of use and efficiency. In both usability tests, quantitative data from the SUS questionnaire (10 questions, each scored from 0 to 4 points) were transformed by multiplying by 2.5 to convert scores to a 0 to 100 range and were categorized adjectivally [42]. Descriptive statistics were analyzed using IBM SPSS Statistics for Windows, version 25.0 (IBM Corp). Both usability tests followed the same semistructured interview guide, comprising 14 open-ended questions. The 5 predefined theory-based app components (ie, self-monitoring, goal setting, coping skills training, social support, and pain education library) were the basis for developing a structured categorization matrix using deductive content analysis [43]. The collected data were coded according to 8 predefined categories, including the 5 components, potential improvements, usage considerations, and coping. Interview responses were transcribed verbatim using NVivo for Windows (QSR International Pty Ltd, version 12, 2018).

### Ethics

The study was approved by the Norwegian Regional Committee for Medical Research Ethics South-East-B (REK reference 2017/350). Participants were informed verbally and in writing that their participation was voluntary, that they could withdraw at any time without a reason (in which case their data would be deleted and destroyed), and that confidentiality and anonymity of their data were ensured at all times. Participants signed informed consent forms before participating.

## Results

### Phase 1: Translation and Cultural Adaptation

The participants did not report having any misunderstanding about or finding discrepancies with the words or phrasing (eg, meaning or activities) of the translated and culturally adapted pain education library, in either usability test. In addition, participant interviews and debriefings in the field usability test (phase 3) were conducted to ensure credibility and understanding of the software interface text. Overall, the participants found the software interface text, which comprised single words and short sentences, easy to understand and interpret, and found the phrasing suitable for their age group.

### Phase 2: Laboratory Usability Test

Participants successfully downloaded the Norwegian version of *the iCanCope with Pain* app and logged in using their mobile phones. After logging in, participants created a mock user profile. They reported finding it easy to perform a daily check-in and liked the idea of monitoring pain patterns, which could contribute to a better understanding of their pain experience. The continuous presence of the avatar figure that changed both face and body expressions according to a numeric scale during registration and feedback made the app easy to use and self-explanatory. However, there were also comments that the profile’s avatar *looked a bit childish*. These participants found it motivating to set goals and read articles in the library section based on those goals. All participants reported that they would recommend the app to others and appreciated the range of pain coping strategies. One participant said:

Hmm, actually it seems like it [the app] has control. So, there is a lot of information. I did not understand at first how an app may help with pain when I first heard about it, but I get it now when I see what it is, yes.

Participants in the laboratory usability test did not make any suggestions regarding how the app could be improved; thus, no adjustments were made before the home-based usability test.

#### User Satisfaction

User satisfaction scores (0 to 100) in the laboratory usability test are shown in Table 1. The average score was 82 out of 100, categorized as *good* and just below *excellent* [42]. The color-based visualization scheme is a modified version of that recommended by Smaradottir et al [44], wherein green represents a positive response, yellow a neutral response, and red a negative response.

**Table 1:** System Usability Scale questionnaire scores from the laboratory usability test.

Questions	P1^a^	P2	P3	P4	P5	P6	Mean (SD)
I think that I would like to use this app frequently	3^b^	3^b^	3^b^	3^b^	4^c^	4^c^	3.3 (0.5)
I found this app unnecessarily complex	2^c^	2^c^	2^c^	1^c^	2^c^	1^c^	1.6 (0.5)
I thought this app was easy to use	5^c^	4^c^	4^c^	4^c^	4^c^	5^c^	4.3 (0.5)
I think I would need assistance to be able to use this app	1^c^	3^b^	1^c^	1^c^	2^c^	4^d^	2 (1.3)
I found the various functions in this app to be well integrated	5^c^	5^c^	4^c^	4^c^	3^b^	5^c^	4.3 (0.8)
I thought there was too much inconsistency in this app	2^c^	2^c^	2^c^	1^c^	1^c^	1^c^	1.5 (0.5)
I imagine that most people would learn to use this app very quickly	4^c^	5^c^	3^b^	5^c^	5^c^	4^c^	4.3 (0.8)
I found this app very cumbersome/awkward to use	1^c^	2^c^	2^c^	1^c^	1^c^	1^c^	1.3 (0.5)
I felt very confident using this app	5^c^	4^c^	3^b^	5^c^	5^c^	4^c^	4.3 (0.8)
I needed to learn a lot before I could get going with this app	1^c^	3^b^	1^c^	1^c^	2^c^	1^c^	1.5 (0.8)
Scores	87.5	72.5	75	90	82.5	85	—^e^
Average	82	—	—	—	—	—	—

^a^Px: participant x.

^b^Neutral response: neither agree nor disagree.

^c^Positive response: agree or strongly agree for positive questions; disagree or strongly disagree for negative questions.

^d^Negative response: agree or strongly agree for negative questions; disagree or strongly disagree for positive questions.

^e^Not applicable.

#### Ease of Use and Efficiency

**Figure 6:**
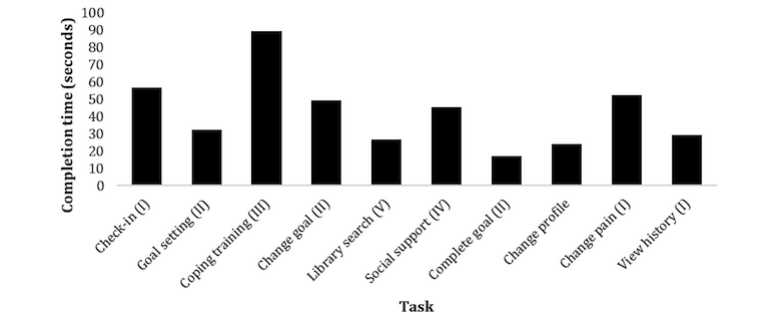
Mean completion time in seconds (0 to 100) for each laboratory usability test task (N=6).

Each participant completed all 10 predefined tasks. As participants progressed through the tasks, some unwanted screen landings or touches were registered as errors. The predefined tasks were completed within the stipulated time frame. Task 3 was expected to be more time consuming as it required the participants to first find a specific article about coping, read the article quietly to themselves, and then read the preferred information bullet points aloud. Efficiency scores are presented in Figure 6 as the mean time in seconds for the completion of each of the 10 predefined tasks related to the 5 app components (I to V).

### Phase 3: Field Usability Test

The daily check-in (ie, self-registration) feature is intended to give users insight into, and an overview of, how they are coping with pain. In total, 4 of the 5 participants used the daily check-in almost every day, primarily after school, with an average of 10.5 check-ins during the 14 testing days. One participant commented, “It [check-in functionality] was a reason for using the app every day” and that “I will miss doing it.” However, 1 participant only used the daily check-in twice and explained in the interview that this was because the app became a reminder of the pain; even positive feedback from the avatar figure *Copey* after a daily check-in was interpreted as negative by this participant, as it was either *too positive* or *just a reminder that I struggled*. Participants created an average of 2.2 goals during the test period. Most goals were related to physical activities, such as participation in soccer practice or burning 200 kcal by running. They also created goals regarding sleep and energy. The participants reported that they appreciated the ability to set goals, said it was a motivating feature, and found it easier to achieve goals when they were written down. The library provided age-appropriate information and pain coping strategies; the participants found this easy to use and interesting, as it offered articles and exercises. One participant reported, “There was a lot of variation in the articles, and I even read about things that I had not related to with my type of pain...” Another participant mentioned that he/she liked using the app in private settings, as he/she did not want to go to a psychologist. Participants favored articles related to CBT, distraction techniques, and help with developing a treatment plan. None of the participants asked for additional help or experienced any technical issues during the test period; thus, no technical issues, help, or user training needs were identified.

#### User Satisfaction

The average user satisfaction score (0 to 100) for the field usability test is shown in Table 2. Participants’ average score was 89, categorized as *excellent* and below *best imaginable* [42].

**Table 2:** System Usability Scale questionnaire scores from the field usability test.

Questions	P1^a^	P2	P3	P4	P5	Mean (SD)
I think that I would like to use this app frequently	4^b^	2^c^	5^b^	3^d^	4^b^	3.6 (1.1)
I found this app unnecessarily complex	1^b^	2^b^	1^b^	1^b^	2^b^	1.4 (0.5)
I thought this app was easy to use	5^b^	5^b^	5^b^	5^b^	5^b^	5 (0)
I think that I would need assistance to be able to use this app	1^b^	1^b^	1^b^	1^b^	1^b^	1 (0)
I found the various functions in this app were well integrated	5^b^	4^b^	5^b^	5^b^	5^b^	4.8 (0.4)
I thought there was too much inconsistency in this app	2^b^	3^d^	1^b^	1^b^	2^b^	1.8 (0.8)
I would imagine that most people would learn to use this app very quickly	5^b^	5^b^	5^b^	5^b^	5^b^	5 (0)
I found this app very cumbersome/awkward to use	1^b^	1^b^	1^b^	1^b^	1^b^	1 (0)
I felt very confident using this app	3^d^	2^c^	5^b^	4^b^	5^b^	3.8 (1.3)
I needed to learn a lot of things before I could get going with this app	1^b^	2^b^	1^b^	2^b^	1^b^	1.4 (0.5)
Scores	90	72.5	100	90	92.5	—^e^
Average	89	—	—	—	—	—

^a^Px: participant x.

^b^Positive response: agree or strongly agree for positive questions; disagree or strongly disagree for negative questions.

^c^Negative response: agree or strongly agree for negative questions; disagree or strongly disagree for positive questions.

^d^Neutral response: neither agree nor disagree.

^e^Not applicable.

#### Sociability

*Sociability* refers to the app’s ability to facilitate user interactions with peers [45]. All participants reported that, in theory, this was a promising idea that would allow them to share their experiences and motivate each other within a social support group. However, only one of the participants made posts to this functionality. This participant explained how this feature could have been improved, including switching to a single chat option with a health care professional (ie, physical therapist), options to create groups with other adolescents who experience similar types of pain, and that questions in the community function should focus on pain coping strategies. No changes were made to refine the app for the upcoming clinical trial on this basis, except to facilitate interaction with peers in the community function.

## Discussion

### Principal Findings

Here, we have described the process of translation and cultural adaptation of the *iCanCope with Pain* app into a Norwegian context, and outcomes of 2 usability tests. Our adolescent study participants did not report having any misunderstanding of or finding discrepancies within the words or phrasing of the translated and culturally adapted app. The laboratory usability tests showed that all 10 predefined tasks were completed within the allocated time frame (ie, were efficient) and were reported to be easy to use. Furthermore, both usability tests showed that the app was self-explanatory, with high satisfaction scores. One home-based usability test participant reported that the app became a reminder of their pain. The community functionality (social support) in the app was rarely used. No technical issues, help, or user training needs were identified.

A 2-stage multistep approach was considered necessary to culturally adapt the app content. The thorough approach used herein may explain why participants found the Norwegian *iCanCope with Pain* app text suitable for their age group, with no discrepancies in phrasing or words. Although several translation and cultural adaptation techniques have been previously described, with different strengths and weaknesses, a transparent and thoroughly described procedure is essential [46]. Nevertheless, 8 steps have been recommended as a minimum when conducting a stepwise translation and adapting instruments intended for a clinical context [47]. In addition, the concept of functional equivalence in cross-cultural research involving adolescents [48] is particularly important; for example, adolescents might engage in different behaviors and understand meanings differently across diverse cultures. Nevertheless, no misunderstandings regarding activities or meanings were reported in this study.

The original Canadian *iCanCope with Pain* app underwent rigorous development and testing through a user-centered design for adolescents with chronic pain, based on their unique health care needs [21]. Furthermore, the *iCanCope with Pain* app is currently under evaluation for use by those with other health conditions, such as arthritis and sickle cells disease [49]. Such preparatory work should be highlighted as it may explain why we failed to identify any technical issues or the need for any additional user assistance or training in either of our usability tests. In addition, this may explain why we found high user satisfaction in both usability tests, with the highest scores among the participants who interacted with the app over time in their natural home environments. These participants reported that they were able to relate specifically to the different app components and thus provided the most valuable feedback from an end-user perspective [50,51].

Despite the participants’ reports that they liked the idea of an app component that would allow them to seek social or peer support, this functionality was rarely used. Nevertheless, research has shown the advantages of peer support delivered via apps, which may provide effective interventions and alleviate stress within other health care systems [52]. Forgeron et al concluded their systematic review by noting that adolescents with chronic pain have peer relationship deficiencies [53]; however, we expect that the rare use of social support in this study was more likely because of our low number of participants. Regardless, social (or peer) support plays a protective role for adolescents with chronic pain and is important for their social development [54].

The app was designed for a generic target group of adolescents with persistent pain originating from different etiologies. Our participants reported appreciating that they were able to access the app from home after school and learn from psychological strategies in the app, which were the most popular articles. Given the free time of adolescents may be limited, measures such as high efficiency (tasks completed within the allocated time frame) and ease of use might be of great importance, by not taking much of the adolescents’ free time. Accessibility of the internet, with options for what, when, and where to read, and creating their own goals could be beneficial for adolescents who might be in a stressful stage of life with school and everyday activities, and for those who may find traditional psychological therapies delivered by adults more difficult [55]. One participant in our study mentioned that he/she did not want to go to a psychologist, possibly reflecting adolescents’ perceived stigma with psychotherapy that has been previously reported [21,55,56]. Mobile phones may have several advantages compared with traditional face-to-face treatments, including their 24/7 availability, pocket size, interactive nature, and flexible programming [57]. However, 1 participant in our study also stated that the app served as a pain reminder and thus was a nonpreferred coping approach. Consistent with this comment, technology and apps for coping may not be suitable methods for empowering all adolescents who experience persistent pain [21].

### Limitations

Several study limitations must be considered. TA was used during tasks to confirm when participants started and ended each of the predefined tasks, providing valuable insight into users’ thoughts and actions [40]. However, not all participants found it natural to verbalize the task as they were performing it, which may have influenced task efficiency because of higher cognitive loads. This may call into question the reliability and validity of these data [58]. Another limitation is that we used convenience sampling of the adolescents, who conducted the translation and cultural adaptation procedure (phase 1) and who served as participants in the laboratory usability test (phase 2). Furthermore, only 2 adolescents were included in phase 1. These adolescents might have related their use of the app in a more hypothetical manner. Ideally, all participants should have been end users, who are known to provide the most valuable feedback [40]. However, recruiting end users was only possible in the final study phase (phase 3) as the first 2 phases were conducted before recruitment for the randomized controlled trial. Participants suggested several potentially valuable improvements that were not feasible. For example, including health care support would make the app a class 2 medical device, and creating groups based on different pain areas was limited by funding and did not correspond with the upcoming trial design. Finally, the app was originally developed and user-tested by groups with a relatively larger age range [21,49] than was used in this study, suggesting that our assessments may not generalize to a larger population. However, our sample was recruited specifically to match the criteria for the upcoming trial, to which they likely generalize.

### Conclusions

This study presented the process of language and cultural adaptation and 2 usability tests for the Norwegian version of the *iCanCope with Pain* app. High user satisfaction, ease of use, efficiency, and only minor errors cumulatively indicated that no changes to the app were needed, with the exception of facilitating user interaction with peers within the social support feature. Despite this, iterative usability testing was fundamental to ensuring that the app is cross-culturally valid and easy to use, before it is used in an upcoming randomized controlled trial with a larger sample.

## Abbreviations

CBT: cognitive behavioral therapy
ISO: International Organization for Standardization
SUS: System Usability Scale
TA: think aloud
UiA: University of Agder
